# Supplementary material for: Catalytic Oxidation of Benzoins by Hydrogen Peroxide on Nanosized HKUST-1: Influence of Substituents on the Reaction Rates and DFT Modeling of the Reaction Path
Source: Molecules. 2023 Jan 11;28(2):747. doi: 10.3390/molecules28020747 (PMC9861975; doi:10.3390/molecules28020747)
Supplement: Supplementary file 1 [file molecules-28-00747-s001.zip › molecules-2065748-supplementary.pdf]

Supporting Information

Catalytic Oxidation of Benzoins by Hydrogen Peroxide on Nanosized HKUST-1: Influence of Substituents on the Reaction Rates and DFT Modeling of the Reaction Path

Darya V. Yurchenko [1], Anton S. Lytvynenko [1,2], Emir N. Abdullayev [1,3], Nina V. Peregon [1], Konstantin S. Gavrilenko [3,4], Alina O. Gorlova [5], Sergey V. Ryabukhin [3,4,5], Dmitriy M. Volochnyuk [3,4,5] and Sergey V. Kolotilov [1,4,*]

1. L. V. Pisarzhevskii Institute of Physical Chemistry of the National Academy of Sciences of Ukraine, Prosp. Nauky 31, 03028 Kyiv, Ukraine
2. Department of Analytical Chemistry, Faculty of Science, Charles University, Albertov 6, 12800 Prague, Czech Republic
3. Enamine Ltd., 78 Chervonotkatska Str., 02094 Kyiv, Ukraine
4. Institute of High Technologies, National Taras Shevchenko University of Kyiv, 60 Volodymyrska Str., 01033 Kyiv, Ukraine
5. Institute of Organic Chemistry of the National Academy of Sciences of Ukraine, 5 Murmanska Str., 02094 Kyiv, Ukraine

* Correspondence: s.v.kolotilov@gmail.com

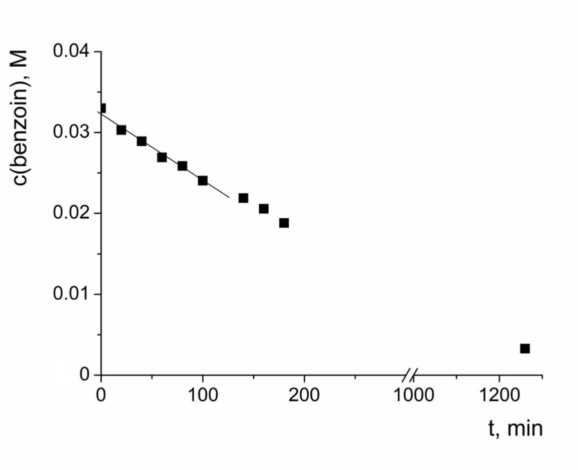

Figure S1. Concentration *vs*. time dependency for the initial period of benzoin oxidation (example curve).

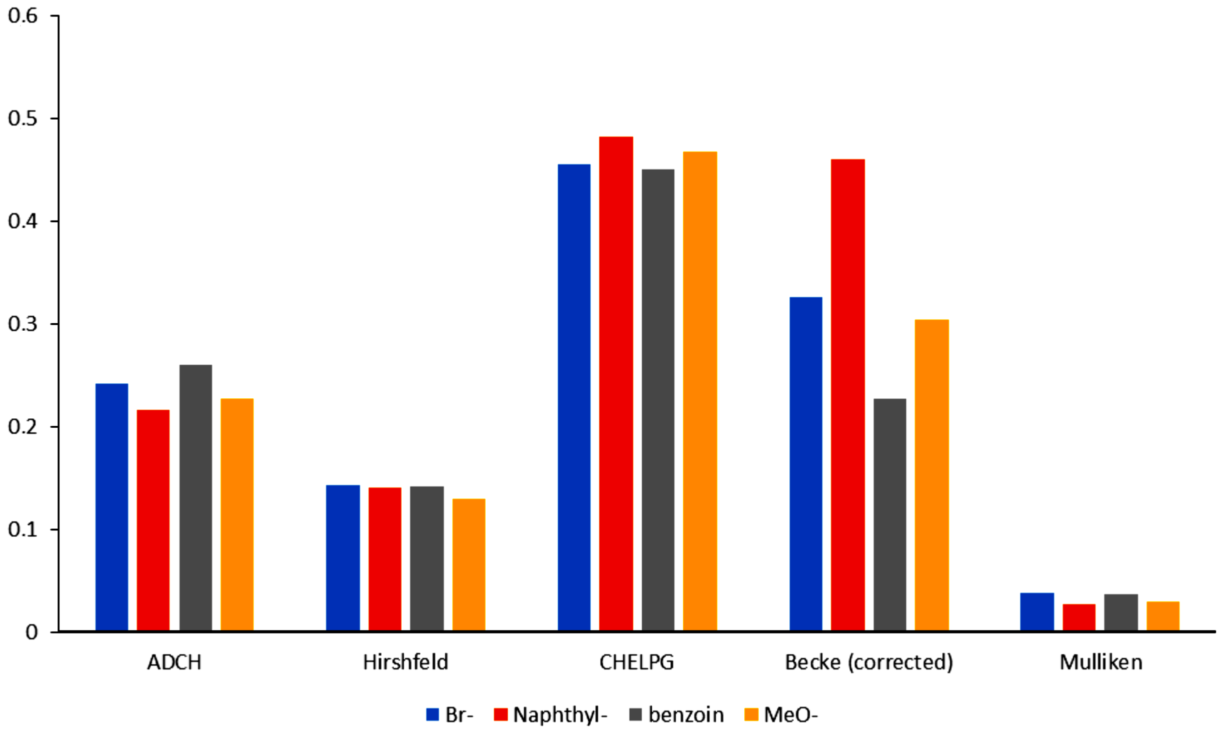

Figure S2. Values of the charges of the carbonyl group's carbon atom of the benzoins studied in this work via different approaches (see Experimental section of the main text for the details).

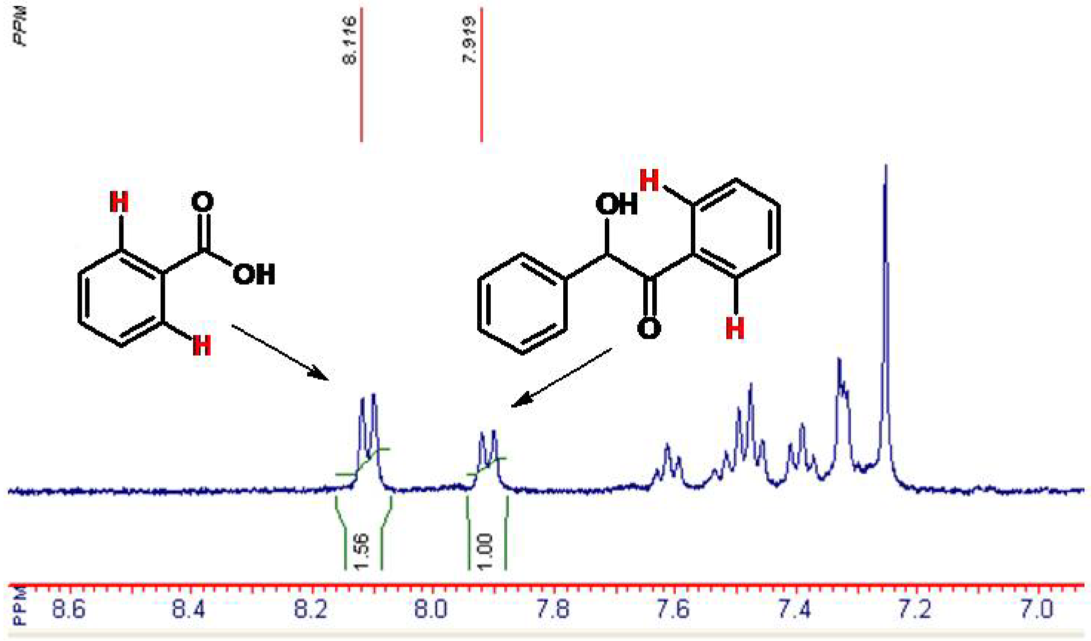

Figure S3. Typical ^{1}H NMR spectrum of the reaction mixture ($CDCl_3$) of the mixture of the starting compound and the product. Arrows point out the signals used for determination of the compounds ratio.

Synthesis of HKUST-1.

Synthesis was performed by electrochemical dissolving of Cu in the electrolyte, containing 320 mg (150 mmol) of H_3btc, 220 mg of Et_4NBF_4 in 10 ml of 96 % ethanol at 60 °C (the temperature was kept during the whole process by a thermostat). Two copper plates of size ca. 13 × 10 mm were used as the electrodes. These plates were thoroughly polished prior to the synthesis with a sandpaper, then cleaned by washing with conc. HNO_3, distilled water and isopropanol. Distance between the electrodes during the electrolysis was 1 cm. Electrolysis was carried out in galvanostat mode. Typical current was 100 mA. The process was stopped when 3 F of electricity per 1 mole of H_3btc passed through a system. The mixture was cooled to a room temperature, the solid was separated by centrifugation, washed several times with warm ethanol with ultrasound treatment and dried on air.

Synthesis of 1,2-bis(2-naphthyl)-2-hydroxyethanone.

10.12 g (65 mmol) of 2-naphthaldehyde were dissolved in 50 ml of ethanol-water mixture (4:1 by volume), then 1.92 g (40 mmol) of freshly crystallized NaCN were added at room temperature. The reaction mixture was heated under refluxed for 0.5 h, then it was cooled to a room temperature. Bright orange precipitate formed. The product was filtered and crystallized from hot isopropanol, and dried in vacuum. Yield 85 %.

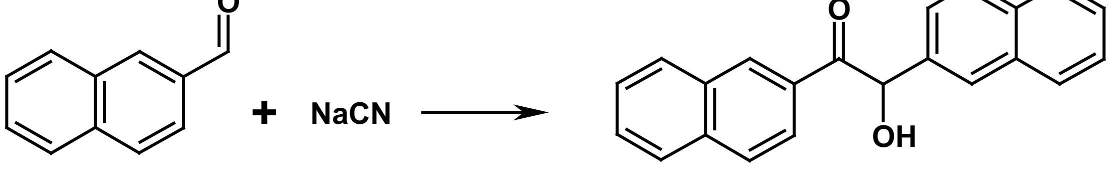

Figure S4. Synthesis of 1,2-bis(2-naphthyl)-2-hydroxyethanone.

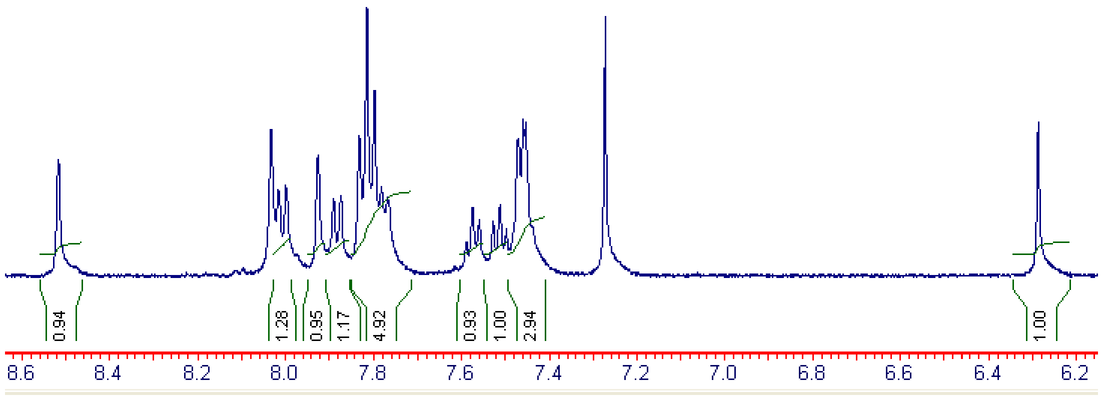

Figure S5. ^{1}H NMR spectrum of 1,2-bis(2-naphthyl)-2-hydroxyethanone in $CDCl_3$.

Synthesis of 1,2-bis(4-methoxyphenyl)-2-hydroxyethanone

10 г (73.5 mmol) of 4-methoxybenzaldehyde and 2 g (31 mmol) of KCN were dissolved in 23 ml of ethanol-water mixture (15:8 by volume) and the solution was heated under reflux for 2 h, then 2 g of KCN were added and heating was continued for 2 h more. The reaction mixture was cooled to a room temperature and was left at intense stirring overnight. Pale yellow crystals of product formed. It was found that long stirring was critically important for the synthesis, else oily product, containing significant quantity of the starting aldehyde, formed instead of the crystals.

The precipitate was filtered and crystallized from hot isopropanol and dried in vacuum. Yield 35 %.

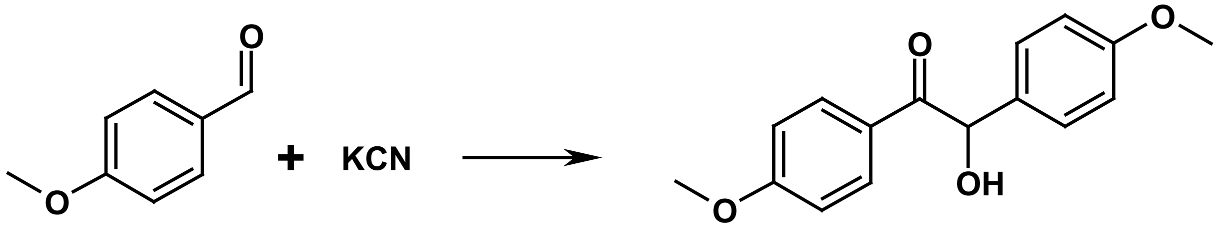

Figure S6. Synthesis of 1,2-bis(4-methoxyphenyl)-2-hydroxyethanone.

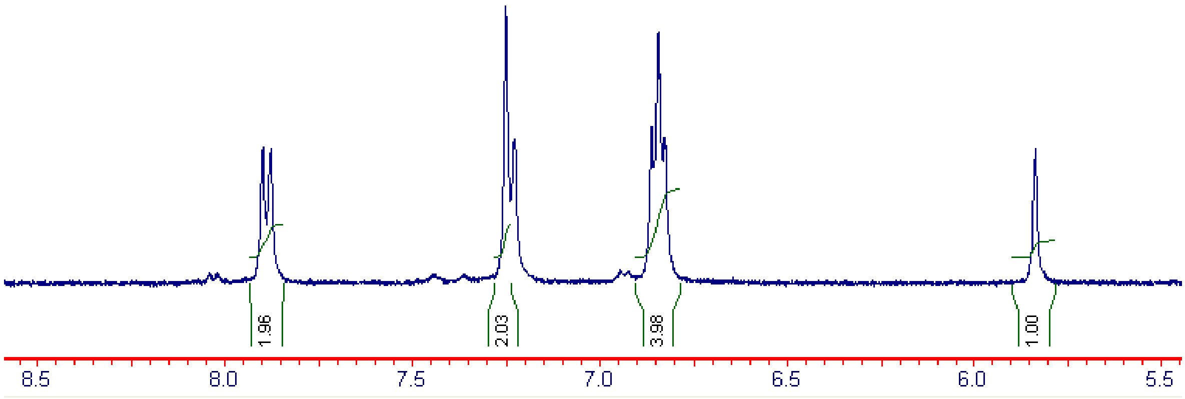

Figure S7. ^1H NMR spectrum of 1,2-bis(4-methoxyphenyl)-2-hydroxyethanone in $CDCl_3$.

Synthesis of 1,2-bis(4-bromophenyl)-2-hydroxyethanone

20 g (110 mmol) 4-bromobenzaldehyde were added to a solution of 1.02 g (21 mmol) of freshly crystallized NaCN in 40 ml of ethanol-water mixture (3:1 by volume), and the reaction mixture was heated under reflux for 2 h. The mixture was cooled down to a room temperature, and pale yellow precipitate formed. The product was filtered and crystallized from isopropanol and dried in vacuum. Yield 80%.

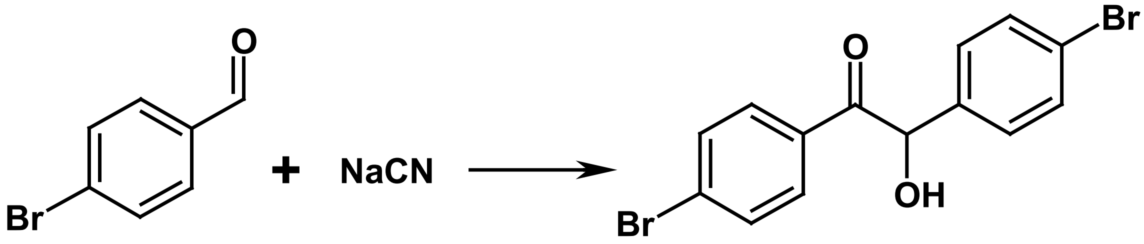

Figure S8. Synthesis of 1,2-bis(4-bromophenyl)-2-hydroxyethanone.

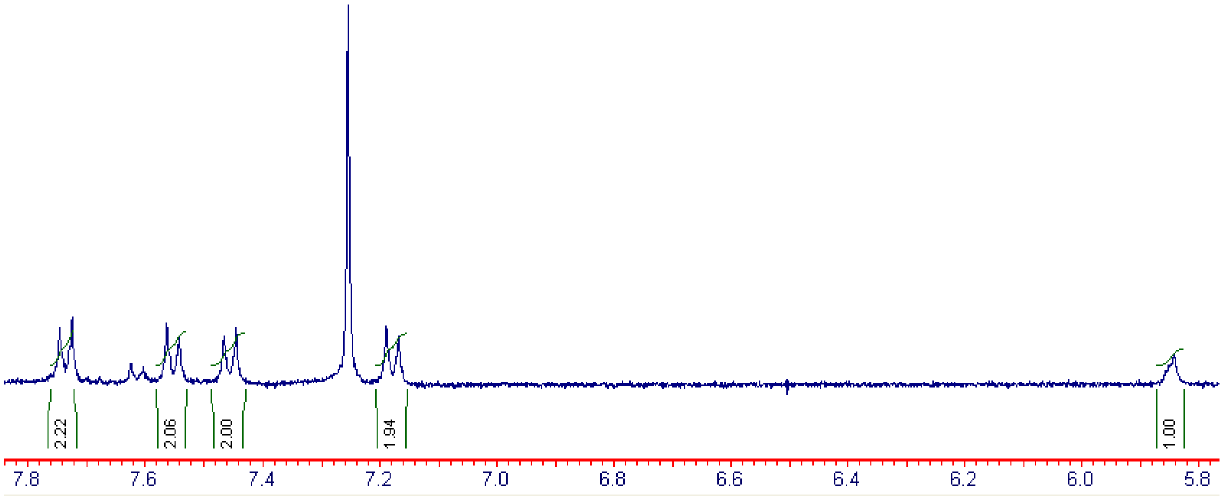

Figure S9. ^{1}H NMR spectrum of of 1,2-bis(4-bromophenyl)-2-hydroxyethanone in $CDCl_3$.

It was found that the result of benzoin condensation significantly depended on the cation in cyanide (NaCN or KCN) and purity of the cyanide. Use on the cyanide, which was not crystallized immediately before the experiment, or the cyanide other than specified above led to significantly lower yields of the products.
